# A new automatic urinometer shows lower bias, no loss of precision due to temporal deviation and higher user evaluation when compared with a manual standard urinometer in an ICU setting

**DOI:** 10.1186/cc13353

**Published:** 2014-03-17

**Authors:** A Eklund, J Van der Linden

**Affiliations:** 1 Karolinska University Hospital, Stockholm, Sweden

## Introduction

In the intensive care setting, most physiologic parameters are monitored automatically. However, urine output (UO) is still monitored hourly by manually handled urinometers. This study evaluated an automatic urinometer (AU) and compared it with a manual urinometer (MU).

## Methods

This was a prospective study in the ICU of a cardio-thoracic surgical clinic. In postoperative patients (*n *= 36) with indwelling urinary catheters and an expected stay of 24 hours or more, hourly UO samples were measured with an AU (*n *= 220, Sippi^®^; Observe Medical, Gothenburg, Sweden) or a MU (*n *= 188, UnoMeter™ 500; Unomedical a/s, Birkeroed, Denmark), and thereafter validated by cylinder measurements. Malposition of instrument at reading excluded measurement. Data were analyzed with the Bland-Altman method. The performance of the MU was used as minimum criteria of acceptance when the AU was evaluated. The loss of precision with the MU due to temporal deviation from fixed hourly measurements was recorded (*n *= 108). A questionnaire, filled out by the ward staff (*n *= 28), evaluated the ease of use of the AU compared with the MU.

## Results

Analysis according to Bland-Altman showed a smaller mean bias for the Au, +1.9 ml, compared with the MU, +5.3 ml (*P *< 0.001). There was no statistical difference in measurement precision between the two urinometers, defined by their limits of agreement (±15.2 ml vs. ±16.6 ml, *P *= 0.11). The mean temporal variation with the MU was ±7.4 minutes (±12.4%), limits of agreement ±23.9 minutes (±39.8%), compared with no temporal variation with the AU (*P *< 0.001). A total 86% of the ward staff considered the AU superior to the MU (*P *< 0.001).

## Conclusion

The AU had a significantly lower bias than the MU and the loss of precision of hourly UO due to temporal deviations using the MU was avoided with the AU. The AU was also evaluated higher by the ward staff, reflecting perception of higher reliability, easier use, less contact with urine bags and less time usage for measurements. The features of the AU may also indicate a favorable clinical impact in the normal ward, when staffing does not allow hourly measurements with a MU.

